# Effect of capsular polysaccharide phase variation on biofilm formation, motility and gene expression in *Vibrio vulnificus*

**DOI:** 10.1186/s13099-024-00620-0

**Published:** 2024-07-29

**Authors:** Tingting Zhang, Shenjie Ji, Miaomiao Zhang, Fei Wu, Xue Li, Xi Luo, Qinglian Huang, Min Li, Yiquan Zhang, Renfei Lu

**Affiliations:** 1 Department of Clinical Laboratory, Nantong Third People’s Hospital, Affiliated Nantong Hospital 3 of Nantong University, Nantong, Jiangsu 226006 China; 2 School of Medicine, Nantong University, Nantong, Jiangsu 226019 China; 3 Department of Clinical Laboratory, Qidong People’s Hospital, Qidong, Jiangsu, 226200 China; 4 Department of Gastroenterology and Clinical Laboratory, Nantong Third People’s Hospital, Affiliated Nantong Hospital 3 of Nantong University, Nantong, Jiangsu 226006 China

**Keywords:** *Vibrio vulnificus*; CPS phase variation, Biofilm, Motility, Regulation

## Abstract

*Vibrio vulnificus*, a significant marine pathogen, undergoes opaque (Op)-translucent (Tr) colony switching based on whether capsular polysaccharide (CPS) is produced. CPS phase variation is sometime accompanied by genetic variation or down-regulation of particular genes, such as *wzb*. In addition, CPS prevents biofilm formation and is important to the virulence of *V. vulnificus*. However, the extent to which there is a difference in gene expression between Tr and Op colonies and the impact of CPS phase variation on other behaviors of *V. vulnificus* remain unknown. In this work, the data have shown that CPS phase variation of *V. vulnificus* is affected by incubation time. Tr and Op strains exhibited similar growth rates. However, Tr strains had enhanced biofilm formation capacities but reduced swimming motility compared to Op strains. The RNA-seq assay revealed 488 differentially expressed genes, with 214 downregulated and 274 upregulated genes, between Tr and Op colonies. Genes associated with Tad pili and CPS were downregulated, whereas those involved in flagellum were upregulated, in Tr colonies compared with Op colonies. In addition, 9 putative c-di-GMP metabolism-associated genes and 28 genes encoding putative regulators were significantly differentially expressed, suggesting that CPS phase variation is probably strictly regulated in *V. vulnificus*. Moreover, 8 genes encoding putative porins were also differentially expressed between the two phenotypic colonies, indicating that bacterial outer membrane was remodeled during CPS phase variation. In brief, this work highlighted the gene expression profiles associated with CPS phase variation, but more studies should be performed to disclose the intrinsic mechanisms in the future.

**Supplementary Information:**

The online version contains supplementary material available at 10.1186/s13099-024-00620-0.

## Introduction

*Vibrio vulnificus* is a Gram-negative, halophilic bacterium commonly found in marine ecosystems that can cause human infections via consumption of seafood or through exposure to seawater [1]. The progress of *V. vulnificus* infection is extremely rapid, and may result in severe consequences without timely treatment, including amputation and death. Globally, the case fatality rate of *V. vulnificus* infection is about 9.1–68.0% [2]. The pathogenic mechanisms of *V. vulnificus* are not fully understood, but its full virulence requires multiple virulence factors, including capsular polysaccharide (CPS), flagella, toxins, proteolytic enzymes, and phospholipase A [2]. Of these, CPS is essential for virulence, as it confers the ability to *V. vulnificus* to resist the killing of the host immune system [2].

There are at least four antigenically different groups of CPS in bacteria. CPS in *V. vulnificus* belongs to the groups 1 and 4, which differ in their sugar residues, assembling model and genetic structures [3, 4]. *V. vulnificus* undergoes phase variation between opaque (Op) and translucent (Tr) colony phenotypes based on CPS production [5]. Tr colonies produce little or no observable CPS, whereas Op colonies contain a mass of CPS [5]. CPS phase variation occurs at both genotypic and phenotypic levels, as observed in a *V. vulnificus* strain with a group 1 CPS operon [6]. Tr strains capable of switching back to the Op form produce reduced amount of CPS as compared to the Op parent and have no mutations within the group 1 CPS operon; by contrast, phase-locked Tr strains have lost the *wzb* gene, which encodes a cognate phosphatase in the group 1 CPS operon, and are thus unable to produce CPS [3, 6]. Additionally, an intermediate (Int) colony phenotype with opacity between that of Op and Tr phenotypes has been observed [7]. Int colonies retain the *wzb* gene but exhibit reduced transcriptional levels and switch to the Tr type frequently, with occasional switches to the Op type [7].

CPS phase variation in *V. vulnificus* is affected by numerous environmental factors, including the amount of oxygen, temperature, and incubation time [3, 5]. Elevated calcium and manganese concentrations up-regulate existing phase variation mechanisms and substantially increase the propensity for Op phenotype to switch to Tr phenotype [8, 9]. The two-component system GacS/GacA is required for CPS phase variation, as the ability of *gacA* mutant to switch to the Tr phenotype was significantly reduced [10]. The sigma factor (σ^S^) is also involved in the regulation of CPS phase variation, as the *rpoS* mutant exhibited significantly higher conversion rates [10]. The master quorum sensing regulator SmcR positively regulates CPS gene expression in *V. vulnificus*, resulting in the *smcR* mutant produced a Tr phenotype on agar plates [11]. Moreover, the periplasmic negative regulator RseB also seems to have a positive regulatory activity on CPS genes, as the *rseB* mutant colonies exhibited a reduced Op phenotype compared to the parental strain [12]. In addition, knockout of the *rfaH* gene, which encodes an antiterminator RfaH protein in an Op strain, led to a diminished capacity to undergo phase variation and little CPS production [13]. Therefore, *V. vulnificus* CPS phase variation is strictly regulated by multiple factors.

CPS phase variation influences the virulence of *V. vulnificus* [2]. CPS production inhibits biofilm formation by *V. vulnificus* [11]. However, it remains unclear whether CPS phase variation affects other behaviors, such as motility, in *V. vulnificus*. In some cases, CPS phase variation is accompanied by genetic variation or the down-regulation of particular genes, such as *wzb* [6]. Therefore, it may be necessary to compare gene expression between Tr and Op colonies to better understand the theoretical basis of CPS phase variation in *V. vulnificus*. In this study, we conducted phenotypic assays focused on biofilm formation and motility, complemented by whole transcriptome sequencing, to delineate the distinctions in biofilm formation and motility, as well as gene expression profiles between Tr and Op colonies.

## Materials and methods

### Bacterial strains

The *V. vulnificus* strain used in this study, named VV2018, was isolated in 2018 from a blood specimen of a clinical patient with hepatitis B virus cirrhosis at Nantong Third People’s Hospital, Nantong, Jiangsu, China [14]. The results of genomic DNA sequencing showed that VV2018 contains a group 1 CPS operon [15]. The genome sequences of VV2018 were deposited in the NCBI GenBank server under the accession numbers CP126698 and CP126699.

### CPS phenotype reversal assay

A pure culture of VV2018 Op colony in Luria-Bertani (LB) broth [1% (w/v) Tryptone (BD Biosciences, USA), 0.5% (w/v) Yeast extract (OXOID, UK) and 1% (w/v) NaCl (Merk, Germany)] was preserved in 40% glycerol and routinely stored at −80 °C. A total of 15 µl glycerol stock of bacterial cells was inoculated into 5 ml LB broth, and incubated at 37 °C with shaking at 200 rpm for 12 h. The resultant cell culture was diluted 50-fold into 5 ml LB broth in glass tubes and incubated statically at 37 ºC for 1 to 13 days. Starting from the first 24 h, randomly selected one glass tube per 24 h, and taken 100 µl of resultant cell culture, 10-fold serially diluted into phosphate buffered saline (PBS) buffer (pH 7.2), and then spread onto an LB plate. The plate was incubated statically at 37ºC for 24 h, observing whether the CPS variation occurs. For each sample, the relative proportions of Tr and Op colonies were determined by calculating their percentages in relation to the total colony count.

### Measurement of growth curves

Growth curves of Tr and Op strains were made similarly as previously described [16]. Briefly, Op and Tr colonies were randomly selected in triplicate from the LB plate, resuspended to PBS, adjusted to an OD_600_ value to 1.4, and then 100-fold diluted into 5 ml of LB broth. The cultures were allowed to continuously grow at 37 °C with shaking at 200 rpm. The OD_600_ values of each culture were measured at 1-hour intervals to create growth curves.

### Crystal violet (CV) staining

CV staining assay was performed as previously described [16, 17]. Briefly, three Op and three Tr colonies were randomly selected from the LB plate, followed by resuspended in PBS buffer. The bacterial suspensions were adjusted to an OD_600_ value of 1.4, which was defined here as bacterial seeds. The bacterial seeds were 50-fold diluted into 1 ml LB broth in a 24-well cell culture plate and allowed to grow at 30 °C with shaking at 100 rpm. The planktonic cells were collected for measurement of OD_600_ values. The surface-attached cells were stained with 0.1% CV, and then dissolved with 20% acetic acid, followed by the measurement of OD_570_ values. Relative biofilm formation was expressed as the values of OD_570_/OD_600_.

### Congo red agar (CRA) assay

CRA assay was performed as previously described [18]. Briefly, a small amount of the bacterial seeds was streaked onto a LB plate containing 0.8 mg/ml of Congo red (Amresco) and 0.4 mg/ml of Coomassie brilliant blue G-250 (Amresco), and then statically incubated at 37 °C for 48 h.

### Swimming motility

Swimming motility assay was performed similarly as previously described [16]. Briefly, 2 µl of bacterial seeds were inoculated into semi-solid swim plates [LB broth supplemented with 0.5% Difco Noble agar (BD Biosciences, USA)]. The swimming diameters were measured per hour after incubation at 37 °C.

### RNA sequencing (RNA-seq)

A total of 15 µl glycerol stock of Op cells was inoculated into 5 ml LB broth, and incubated at 37 °C with shaking at 200 rpm for 12 h. The resultant cell culture was diluted 50-fold into 5 ml LB broth in a glass tube and incubated statically at 37 °C for 4 days. A total of 100 µl resultant cell culture was taken, diluted serially with PBS, spread onto an LB plate, and then incubated statically at 37 °C for 24 h. Thereafter, three Op and three Tr colonies were randomly collected from the LB plate using bacteria-free toothpicks, respectively, and then dissolved in TRIzol reagent (Invitrogen, USA) for RNA extraction [16]. RNA quantity was measured using a Nanodrop 2000 (Thermo Fisher Scientific, USA). The amount of total RNA in each sample was at least 2 µg with an OD_260_/OD_280_ value between 1.8 and 2.2. RNA integrity number (RIN) was evaluated using an Agilent 2100/2200 Bioanalyzer (Agilent Technologies, USA) with RIN > 6.5 considered good integrity. The rRNA removal and mRNA enrichment was performed using an Illumina/Ribo-Zero™ rRNA Removal Kit (bacteria) (Illumina, USA). All RNA-associated manipulations were performed in GENEWIZ Biotechnology Co. Ltd. (Suzhou, China). Sequencing of cDNA library, which was constructed by using a QIAseq FastSelect-5 S/16S/23S kit (Bacteria) (QIAGEN, Germany), was performed on an Illumina Hiseq platform [16].

Raw data of RNA-seq was filtered by Cutadapt to remove adapters, contamination and low quality reads (v1.9.1) [19]. Alignment of filtered reads was performed using Bowtie2 (v2.2.6) with *V. vulnificus* VV2018 as the reference genome [20]. Gene expression in Tr colonies (test groups) were compared with that in Op colonies (reference group). HTSeq (v0.6.1) and FPKM (Fragments Per Kilo bases per Million reads) methods were applied to calculate gene expression [21, 22]. DESeq (v1.6.3) of the Bioconductor package was used to analyze the difference in gene expression between test and reference groups with selection criteria of pvalue (fdr, padj) < = 0.05 and absolute fold change (|log_2_ FC|) > = 1.5 [23]. Gene Ontology (GO) functional annotation was performed to analyze the significantly differently expressed genes (DEGs) involved in molecular functions, cellular components, and biological processes [24]. Main metabolic pathways of DEGs were analyzed by the Kyoto Encyclopedia of Genes and Genomes (KEGG) pathway enrichment analysis [25]. The functions of proteins encoded by DEGs were predicted by the Cluster of Orthologous Groups of proteins (COG) database [26].

### Quantitative PCR (qPCR)

The qPCR assay was performed similarly as previously described [16, 27]. Briefly, total RNA was extracted from Op and Tr colonies using TRIzol reagent (Invitrogen). cDNA was generated from 1 µg total RNA using a FastKing First Strand cDNA Synthesis Kit (Tiangen Biotech, China). Relative mRNA levels of each target gene were determined using the classic 2^−ΔΔCt^ method [27]. The 16 S rRNA gene was used as the internal control. Primers used for qPCR are listed in Table 1.

**Table 1:** Primers used for qPCR

Target	Primers (forward/reverse, 5’-3’)
1-1244	AACCCGAGTTAGCCAAGCA/GAGTAATCACACGCGCCCCAA
1-1282	CGCCGATGTTCTACCCATGCAA/CGGCAACGGATCACTGAACCA
1-1665	CGTCTTACTCACTGCACGTA/GTGCCTGAGTAATCAATACCAA
1-2669	ATCAAGGCAACTTCGCAGCAA/CGCCATCTGTTTCGCAAGCTC
2–27	TCTGCTGGCAAATCCCGAC/CCTTACCTGCGTTTAGTACAGAC
2–53	TACATTAAAGGTGTATTTGGTGC/TCATTAATCGTCTCGGGAGC
2–65	CCTCAGCGTCTTTGACACTCC/ATTTTGCGTCCATCTAGACCA
2-236	AATACAGACTTAGCGATCACC/ATTACCATTTTCAGAGCTCAA
2-243	GCACCTCCATCTCTATTCGT/GCGATAGACAACAACTTGGC
2-1283	CCAATTACGCTGGCCGACA/ATCCGTGATTTCAAACGTGGT

### Replicates and statistical methods

Phenotypic experiments and qPCR assays were performed independently at least three times, each with three replicates, and the values were expressed as the mean ± standard deviation (SD). A paired Student’s *t*-test was employed to calculate statistically significant differences, with *p* < 0.01 considered significant.

## Results

### Conversion rate of CPS phase variation

**Fig. 1:**
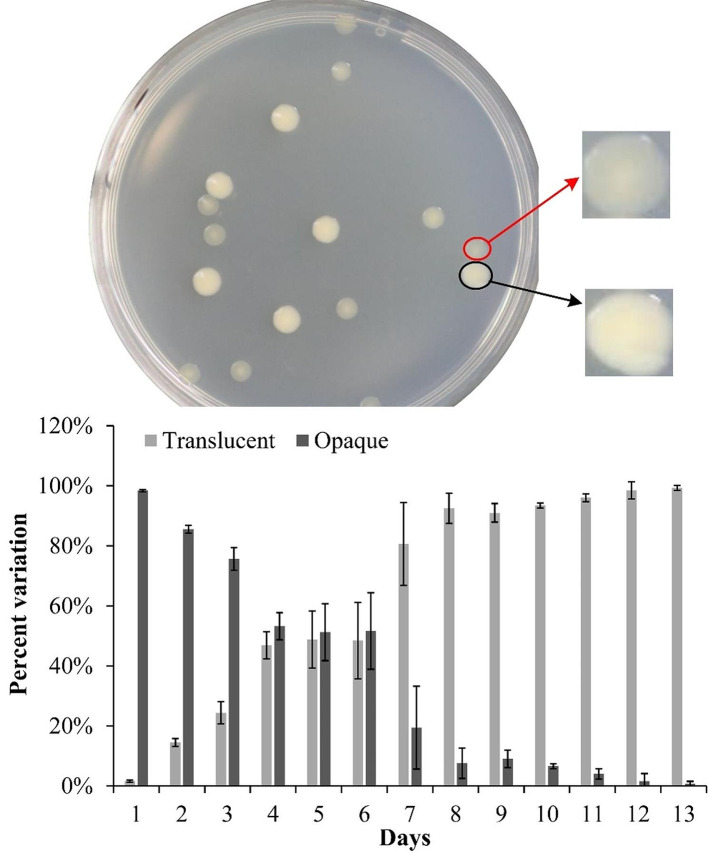
Rate of the CPS variation of *V. vulnificus*. *V. vulnificus* VV2018 was statically incubated in LB broth at 37ºC for 1 to 13 days, transferred to agar plates every 24 h, and incubated for an additional 24 h at 37 °C. The experiments were performed three times, with three plates used for each time point in each trial. The photograph at the top was photographed at the 7 th day. The images indicated by arrows are enlarged for clarity

*V. vulnificus* were cultured statically in LB broth at 37 °C to investigate the conversion rate of Op and Tr phenotypes over time. As shown in Fig. 1, almost all the colonies were Op during the first 24 h. However, the proportion of Op colonies gradually decreased with the increasing of incubation time, while that of Tr colonies gradually increased. On the 7th day, the proportion reversed, and by the 13th day, almost all the colonies were transparent. These results suggested that the incubation time affects CPS phase variation of *V. vulnificus*. However, *V. vulnificus* grows very fast, so with long time incubation, the nutrients would be exhausted and the bacterium would be in the death phase. As shown in Figure S1, the number of bacterial cells (both Op and Tr types) peaked during the first three days and then continued to decrease, indicating that by the third day, the nutrients were likely exhausted, and the bacteria had entered the death phase. Although the total number of bacterial cells decreased with the passage of incubation time, the increase in the proportion of Tr cells was generally equivalent to the decrease in that of Op cells, suggesting that the increase in conversion rate could not be simply attributed to the death of Op cells or the growth of Tr cells. It is still unclear whether the rise in the proportion of Tr cells is due to phase variation or the death of Op cell variants.

### Growth of Op and Tr strains

**Fig. 2:**
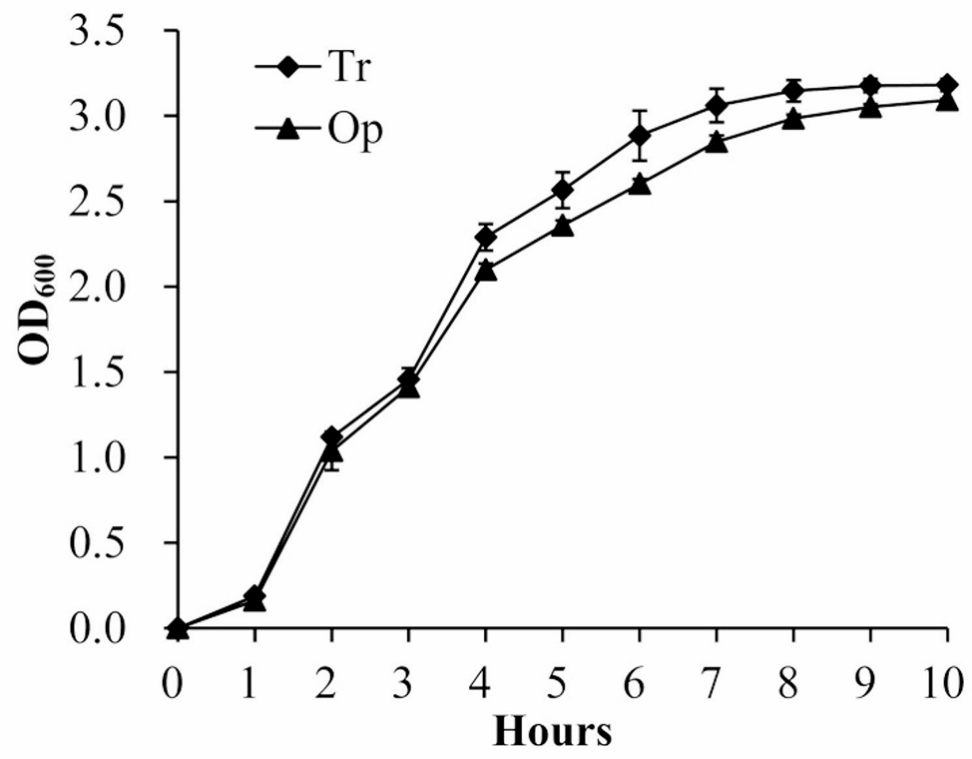
Growth curves of Op and Tr strains. *V. vulnificus* strains were grown in LB broth at 37 °C with shaking at 200 rpm, and the OD_600_ values of each culture were measured at 1-hour intervals. The experiments were performed three times, with three colonies used per trial

The growth curves of Op and Tr strains were measured to determine whether CPS phase variation affects the growth of *V. vulnificus*. As shown in Fig. 2, bacterial cells from the Op colonies exhibited a similar growth rate to those from Tr colonies in LB broth at 37 °C. In addition, 100 µl of growth culture was sampled from the adaptation (1 h), logarithmic (3 and 5 h) and stationary phase (8 and 10 h), respectively, and then spread onto an LB plate to check for colonial reversion. The results showed that Tr strains of *V. vulnificus* did not revert to the Op form, and similarly, Op strains did not switch to the Tr form, throughout the entire observed growth curves (data not shown). Thus, the growth conditions and incubation time might affect the transition between Tr and Op phenotypes, as a significant transition was observed after 2 days of static cultivation (Fig. 1). In conclusion, these results suggested that CPS phase variation did not affect the growth of *V. vulnificus*.

### Tr strains had stronger biofilm formation capacities than Op strains

**Fig. 3:**
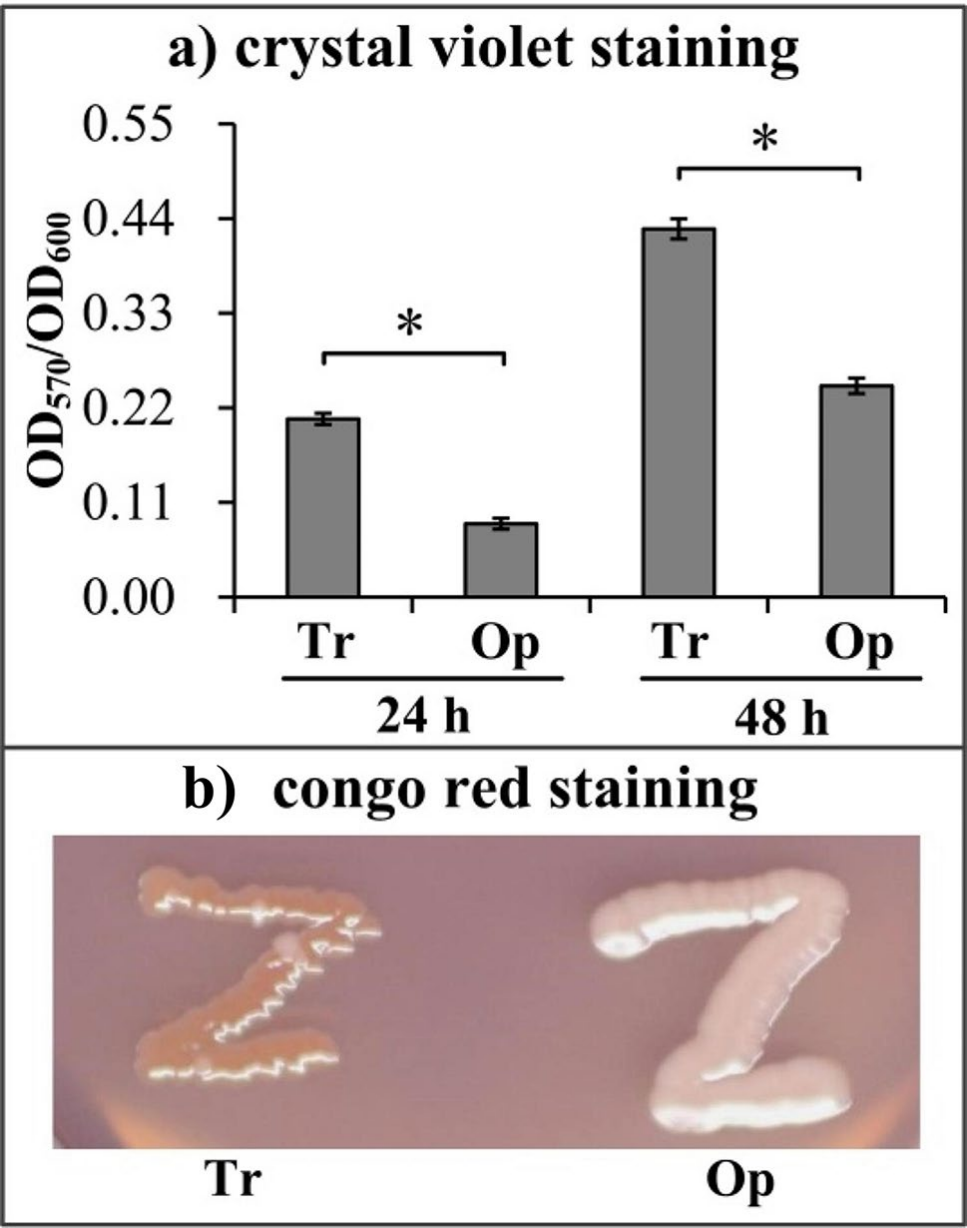
Tr strains had stronger biofilm formation ability than Op strains. Biofilm formation by Op or Tr strains was assessed using crystal violet staining (**a**) and Congo red agar analysis (**b**). Each type of experiment was performed at least three independent times, with three replicates for each time. The numerical values were expressed as the mean ± standard deviation (SD). The photographs are representative of three independent experiments, each with three replicates. The asterisks indicate statistical significance (*p* < 0.01) as assessed by a paired Student’s *t*-test

CPS phase variation is directly associated with the production of CPS, which plays a critical role in inhibiting attachment and biofilm growth [11, 28]. As expected, Tr strains produced more biomass than Op strains in the CV staining-based biofilm formation assay at all time points tested (Fig. 3a). As further verified by the CRA assay (Fig. 3b), Tr strains were able to pick up CR dye from the CRA, resulting in red colonies, whereas Op strains, which produced a mass of CPS, and were incapable of enriching CR dye, resulting in white colonies. These results suggested that Tr strains had a stronger capacity for biofilm formation than Op strains.

### Tr strains had weaker motor ability than Op strains

**Fig. 4:**
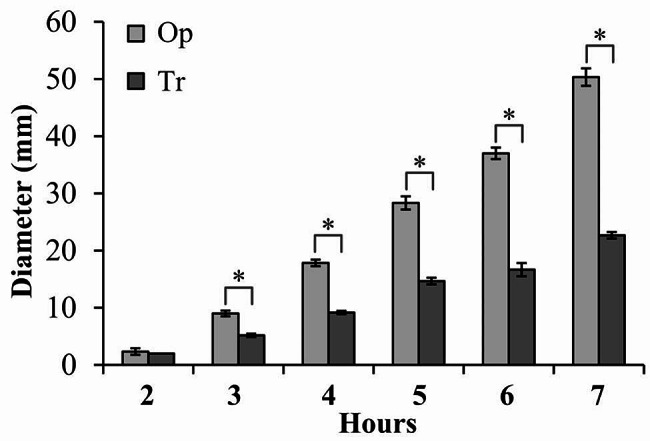
Swimming capacities of Tr and Op strains. Swimming abilities of Tr and Op strains were assessed by measuring the diameters of bacterial swimming areas in semi-solid swimming plates. The experiments were performed three independent times with three replicates each, and the values were expressed as the mean ± standard deviation (SD). The asterisks indicate statistical significance (*p* < 0.01) as assessed by a paired Student’s *t*-test

Flagellum-mediated motility is involved in initial stages of biofilm formation [29]. The difference in biofilm formation capacities between Tr and Op phenotypes promoted us to detect whether they also differed in motor capacity. As shown in Fig. 4, starting from the third hour after cultivation, the swimming motility of Tr strains was significantly lower compared to Op strains, and the diameters of bacterial motility areas for both phenotypes remarkably increased over incubation time. These results suggested that Tr strains had a weaker motor ability than Op strains.

### Screening DEGs between Tr and Op strains through RNA-seq

**Fig. 5:**
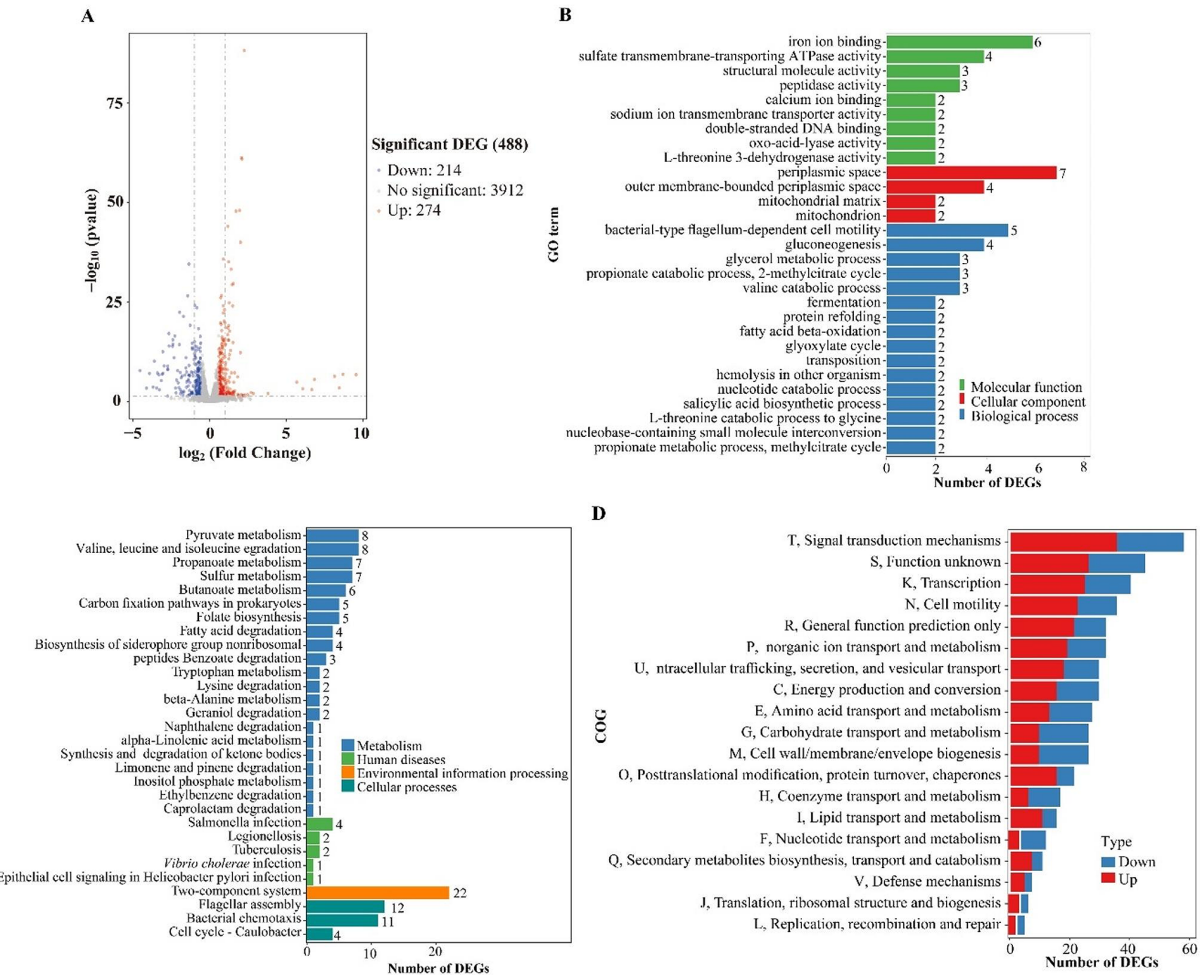
Gene expression of Op and Tr colonies. Three colonies of Op and three of Tr were randomly selected from a plate to obtain total RNA for RNA-Seq analyses. (**a**) Volcano plot. Red, blue, and grey points represent the up-regulated, down-regulated and no-significant changed genes, respectively. (**b**) The enrichment of gene ontology (GO) term. Green, red, and blue bars represent molecular function, cellular component, and biological process, respectively. The number on the right of each bar indicates the number of enriched DEGs. (**c**) Kyoto Encyclopedia of Genes and Genomes (KEGG) enrichment. Blue, green, yellow, and blue-green bars represent metabolism, human diseases, environmental information processing, and cellular process, respectively. (**d**) Cluster of Orthologous Groups of proteins (COG) enrichment. Red and blue bars represent the up- and down-regulated DEGs, respectively

The mRNA profiles in Tr colonies (test) were compared with those in Op colonies (reference) by RNA-seq to analyze the genes responsible for phenotypic changes. We sequenced a total of six Illumina libraries, obtaining over 14.5 million clean reads for each, with more than 95% uniquely mapped to the genome of *V. vulnificus* VV2018. A total of 488 genes were significantly differentially expressed in Tr colonies relative to Op colonies, with 214 downregulated and 274 upregulated (Fig. 5a; Table 2). The results of GO enrichment showed that DEGs were enriched into biological process (16 GO terms, 40 DEGs), followed by molecular function (9 GO terms, 26 DEGs) and cellular component (4 GO term, 15 DEGs) (Fig. 5b). The results of KEGG enrichment showed that 72 DEGs were involved in metabolism, 10 DEG was involved in human diseases, 22 DEGs were involved in environmental information processing, and 27 DEGs were involved in cellular processes (Fig. 5c). The results of COG enrichment showed that DEGs were divided into 19 functional categories, with the top five enrichment pathways being signal transduction mechanisms, function unknown, transcription, cell motility, and general function prediction only (Fig. 5d).

**Table 2:** DEGs in Tr colonies relative to Op colonies

Gene		Fold change	Product
VV2018	CMCP6		
**Tad pili**			
2_239	E2I22_21420	0.0774	pilus assembly protein CpaB
2_240	E2I22_21425	0.1001	General secretion pathway protein GspD
2_241	E2I22_21430	0.1275	Hypothetical protein
2_242	E2I22_21435	0.0936	Type II/IV secretion system ATPase TadZ/CpaE, Associated with Flp pilus assembly
2_243	E2I22_21440	0.0815	CpaF family protein
2_244	E2I22_21445	0.1123	pilus assembly protein TadB
2_245	E2I22_21450	0.0906	type II secretion system F family protein
2_246	E2I22_21455	0.1199	Tetratricopeptide repeat protein
2_247	E2I22_21460	0.1492	Pilus assembly protein
2_248	E2I22_21465	0.2316	Pilus assembly protein TadF
2_249	E2I22_21470	0.1510	Pilus assembly protein TadG
**CPS synthesis**			
2_59	E2I22_20505	0.2660	glycosyltransferase
2_60	E2I22_20510	0.0570	capsular biosynthesis protein
2_61	E2I22_20515	0.2484	glycosyltransferase
2_62	E2I22_20520	0.5665	polysaccharide biosynthesis tyrosine autokinase
2_64	E2I22_20530	0.3042	capsular biosynthesis protein
2_65	E2I22_20535	0.2248	Undecaprenyl-phosphate glucose phosphotransferase
**Flagellum**			
1_701	E2I22_03510	1.5230	flagellar basal body rod protein FlgB
1_702	E2I22_03515	1.5456	flagellar basal body rod protein FlgC
1_703	E2I22_03520	1.6046	flagellar hook assembly protein FlgD
1_704	E2I22_03525	1.5101	flagellar hook protein FlgE
1_705	E2I22_03530	1.5892	flagellar basal-body rod protein FlgF
1_706	E2I22_03535	1.5189	flagellar basal-body rod protein FlgG
1_707	E2I22_03540	1.5258	flagellar basal body L-ring protein FlgH
1_708	E2I22_03545	1.5776	flagellar basal body P-ring protein FlgI
1_710	E2I22_03555	1.6208	flagellar hook-associated protein FlgK
1_711	E2I22_03560	1.5904	flagellar hook-associated protein FlgL
1_712	E2I22_03565	1.5865	flagellin
1_919	E2I22_05085	1.7523	OmpA family protein
1_2076	E2I22_11590	1.5144	flagellar hook-length control protein FliK
**Putative c-di-GMP-associated proteins**			
1_6	E2I22_16100	1.5280	EAL domain-containing protein
1_1181	E2I22_06450	1.9588	GGDEF domain-containing protein
1_1739	E2I22_10100	1.9566	EAL domain-containing protein
1_1857		2.2418	GGDEF domain-containing protein
1_1963	E2I22_10890	1.5805	GGDEF domain-containing protein
2_291	E2I22_21690	1.6914	EAL domain-containing protein
2_1266	E2I22_18895	1.7330	GGDEF domain-containing protein
2_1323	E2I22_19175	0.5206	GGDEF domain-containing protein
2_1521	E2I22_20205	1.8637	EAL domain-containing protein
**Putative porin**			
1_722		0.4481	Porin
1_1104	E2I22_06065	0.5767	Porin
1_1939	E2I22_10775	0.3748	Porin
1_2009	E2I22_11240	0.5195	Aquaporin Z
1_2620	E2I22_14570	0.2612	Porin
2_650	E2I22_23610	0.3896	OmpA family protein
2_669	E2I22_23705	1.6972	Porin
2_671	E2I22_23715	1.7116	Porin
**Putative regulators**			
1_116	E2I22_00395	1.5111	Transcriptional regulator OmpR
1_1244	E2I22_06780	3.2699	Putative HTH-type transcriptional regulator YdfH
1_1282	E2I22_06980	0.3403	LuxR family transcriptional regulator
1_1420	E2I22_07595	0.6375	Response regulator
1_1722	E2I22_10010	0.6588	Response regulator
1_1843		746.5614	Helix-turn-helix domain-containing protein
1_2020	E2I22_11300	0.6183	Transcriptional regulator
1_2584	E2I22_14390	1.5109	Predicted transcriptional regulator
1_2678	E2I22_14925	0.6588	Response regulator transcription factor
1_338	E2I22_01415	1.9514	Transcriptional regulator LeuO
1_457	E2I22_02020	0.5837	Sigma-54 dependent transcriptional regulator
1_533	E2I22_02425	1.8264	Predicted transcriptional regulator
2_1048	E2I22_17805	2.3249	Response regulator
2_1172	E2I22_18420	0.5068	LuxR family transcriptional regulator
2_1226	E2I22_18705	1.6024	Response regulator
2_1285		1.7877	Crp/Fnr family transcriptional regulator
2_1292	E2I22_19025	1.5731	HTH-type transcriptional regulator MalT
2_1322	E2I22_19170	0.6264	Response regulator
2_1389	E2I22_19495	0.6138	AraC family transcriptional regulator
2_1431	E2I22_19695	0.5282	Helix-turn-helix transcriptional regulator
2_1452	E2I22_19800	0.6207	Transcriptional regulator
2_236	E2I22_21405	0.3046	LysR family transcriptional regulator
2_257	E2I22_21525	0.6455	Response regulator
2_27	E2I22_20345	2.7373	MerR family transcriptional regulator
2_487	E2I22_22785	1.8690	Transcriptional regulator
2_53	E2I22_20475	0.3792	Helix-turn-helix transcriptional regulator
2_591	E2I22_23315	1.6317	MerR family DNA-binding transcriptional regulator
2_942	E2I22_17280	1.9747	LysR family transcriptional regulator

### Validation of RNA-seq data by qPCR

**Fig. 6:**
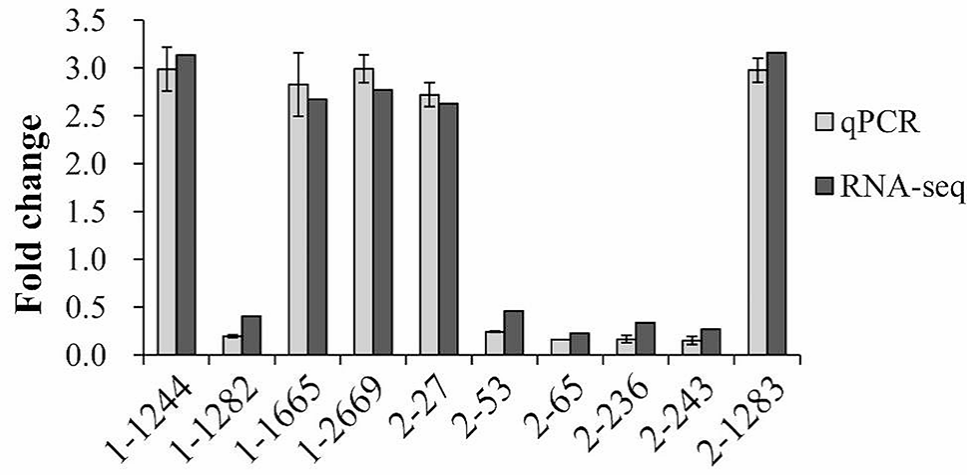
Validation of RNA-seq data by qPCR. The relative mRNA levels of each target gene were compared between Op and Tr colonies to identify differences in expression. The 16 S rRNA gene was used as an internal control for normalization. The experiments were performed three times, with each trial involving independent RNA preparations and three replicates each. Relative mRNA levels of each target gene were determined using the classic 2^−ΔΔCt^ method

The qPCR assay was used to validate the RNA-seq data. Ten DEGs were selected as target genes (Table 1), and the results showed that the expression levels of all the tested genes were consistent with the RNA-seq findings (Fig. 6), confirming the reliability of the transcriptome data.

## Discussion

*V. vulnificus* exhibits the phase variation between Op and Tr morphologies, which is directly associated with whether CPS expression or not [3, 6]. Previous studies have demonstrated that CPS-associated phase variation is influenced by incubation time and environmental factors, including aeration, temperature and the presence of metal ions such as manganese and calcium [3, 5, 9]. Anaerobic microenvironments formed by static growth in test tubes remarkably affect the occurrence of bacterial morphology diversity [30]. In addition, CPS expression varies with the growth phase, increasing during log-phase growth and declining in stationary culture [31]. The data presented here also show that incubation time affects CPS phase variation of *V. vulnificus* (Fig. 1). However, the extent to which the culture media influence the CPS phase variation requires further investigation.

CPS expression inhibits attachment and biofilm formation [28]. It plays a critical role in determining biofilm size by restricting the growth of mature biofilms [11]. The data showed that the Tr strains had stronger biofilm formation but weaker motor capacities than Op strains (Figs. 3 and 4). In addition, Tr and Op strains had no difference in growth rate, indicating that the differences in biofilm formation capacity between the two phenotypes were not associated with their growth rates. Flagellum-mediated motility is involved in initial stages of biofilm formation and the development of biofilm architecture [29]. The loss of flagella hinders bacterial attachment to the surface and biofilm development [32–35]. However, the influence of flagellum-mediated motility on biofilm formation seems to extend beyond attachment and biofilm development, the other roles of flagella and motility in biofilm formation deserve further investigation.

CPS phase variation affects the transcription of 488 genes (Fig. 5a and Table S1). Of these, 6 genes associated with CPS biosynthesis were down regulated in Tr colonies compared to Op colonies, suggesting that the CPS phase variation is accompanied by transcriptional differences in CPS genes between the two phenotypes. No difference in transcriptional levels of *wza* (1_208), *wzb* (1_2108) and *wzc* (1_211) between the two phenotypes was observed (data not shown). Deletion mutations in *wzb* or multiple genes in the group 1 CPS operon were locked in the Tr state [8]. The transcripts of all the group 1 CPS genes in the Tr colonies used for RNA-seq were detected, and thus the CPS phase variation in these strains might be reversible. However, it remains challenging to clearly explain why CPS phase variation influences the transcription of numerous genes. Perhaps behind the surface of CPS phase variation lies a complex and tightly regulated processes of bacterial metabolism and gene expression. In addition, bacterial DNA methylation has been demonstrated to be involved in many physiological activities, including chromosome replication, DNA degradation, and gene expression regulation [36]. Whether CPS phase variation is accompanied by DNA methylation and thereby affecting gene expression needs further exploration.

*V. vulnificus* genome harbors three distinct *tad* gene clusters encoding type IV Tad pili [37]. The *tad-1* gene cluster does not play any role in the virulence of *V. vulnificus* [37]. The *tad*-2 gene cluster is induced in artificial seawater [38]. The *tad*-3 gene cluster is involved in initial surface attachment, auto-aggregation, biofilm formation, and oyster colonization [39]. The three *tad* loci work coordinately, as any one gene cluster can partially complement the phenotypic changes of the *tad* triple mutant, which exhibits a significantly decrease in virulence and biofilm formation, and delayed RtxA1 exotoxin secretion compared to the wild type [40]. The RNA-seq data showed that only *tad-2* genes were significantly repressed in Tr colonies compared with Op colonies (Table 2), suggesting that one of the mechanisms of CPS phase variation affecting biofilm formation may be mediated by altering the expression levels of Tad pili. However, role of Tad pili in the CPS phase variation needs to be further investigated. In addition, the transcription levels of 13 of flagellar genes were significantly enhanced in the Tr colonies (Table 2), but Tr strains had weaker swimming ability than Op strains (Fig. 4), which might be due to the distinct growth conditions applied in RNA-seq versus the swimming motility assay. Specifically, the bacterial cells used for the motility assay were effectively undergoing a re-culturing process, which was not the case for those subjected to RNA-seq. Moreover, 9 genes encoding EAL or GGDEF domain-containing proteins were significantly differentially expressed in Tr colonies relative to Op colonies (Table 2). The GGDEF domain possesses the diguanylate cyclase (DGC) activity, whereas the EAL domain has the phosphodiesterase (PDE) activity, which are responsible for the biosynthesis and degradation of bis-(3’-5’)-cyclic di-GMP (c-di-GMP), respectively [41]. c-di-GMP regulates multiple bacterial behaviors including motility and biofilm formation [41], but there is no significant different in c-di-GMP levels between the two phenotypes (data not shown). Therefore, further research is needed to determine whether these genes are active DGC or PDE in *V. vulnificus*.

The RNA-seq data also revealed that the transcriptional levels of 28 genes encoding putative regulators were significantly differentially expressed in Tr colonies compared to Op colonies (Table 2). Of these, 1_1282 and 2_1172 encode LuxR family transcriptional regulators; 2_37 and 2_591 encode MerR family transcriptional regulators; while 2_236 and 2_942 encode LysR-type transcriptional regulators. The LuxR and LysR family transcriptional regulators are global regulators that control the expression of a variety of genes, including those involved in virulence, motility, and biofilm formation [42, 43]. The MerR family regulators activate transcription in response to environmental stimuli, such as oxidative stress, metal ions or antibiotics [44]. Moreover, 1_116 and 1_338 encode OmpR and LeuO, respectively. *V. vulnificus* LeuO represses the transcription of its own gene and *vvpS* encoding a serine protease, but enhances the expression of Huαβ [45, 47]. Roles of OmpR in *V. vulnificus* have not being investigated, but *V. cholerae* OmpR was shown to be involved in controlling the virulence and fitness [48, 49]. In short, the functions of the vast majority of these 28 putative regulators remain unknown. To further clarify their functions will help us understand the regulation mechanisms of CPS phase variation.

A total of 8 genes encoding putative porins were significantly differentially expressed in Tr colonies compared to Op colonies, of which 2 (2_668 and 2_671) was upregulated and the other 6 (1_722, 1_1104, 1_1939, 1_2009, 1_2620 and 2_650) were downregulated (Table 2). These results suggested that the major outer membrane proteins should be remodeled during the CPS phase variation. In addition, a gene (2_26), which encodes the protein containing the domain of unknown function 523 (DUF523) and DUF1722, was upregulated in Tr colonies compared to Op colonies (Table S1). DUF1722 is homologous to YbgA from *E. coli*, while DUF523 is involved in the modification of RNA species via conversion of 2-thiouracil into uracil [50]. Roles of 2_26 deserve investigate in the future. In addition, 1_1843 encoding a helix-turn-helix domain-containing protein was upregu-lated 746.5614-fold in Tr strain relative to Op strain. The helix-turn-helix motif is usually found in transcriptional regulators [51]. Therefore, it is essential to explore the regulatory roles of 1_1843, particularly its influence on CPS phase variation and biofilm formation, in future studies.

In conclusion, our findings demonstrated that the *V. vulnificus* CPS phase variation was affected by incubation time. Tr and Op strains manifested similar growth rates. However, Tr strains had stronger biofilm formation capacity but weaker swimming motility than Op strains. The RNA-seq data showed that 488 genes were differentially expressed between the two phenotypic colonies, including *tad* pili genes, CPS-associated genes, flagellar genes, c-di-GMP metabolism-related genes, porin genes and regulator encoding genes. Genes involved in the biosynthesis of Tad pili and CPS were downregulated in the Tr colonies, whereas those involved in flagellum were upregulated. However, transcriptome analysis is only a preliminary research of the mechanisms associated with CPS phase variation, and more studies should be performed to disclose the molecular mechanisms involved in *V. vulnificus* CPS phase variation in the future.

### Data availability

The original data presented in the study are included in the article/supplementary materials. Further inquiries can be directed to the corresponding authors. The raw data of RNA-seq have been deposited in the NCBI repository under accession number PRJNA982607.

### Electronic supplementary material

Below is the link to the electronic supplementary material.

Supplementary Material 1

Supplementary Material 2

## Data Availability

The raw data of RNA-seq have been deposited in the NCBI repository under accession number PRJNA982607.
