# Supplementary material for: Effect of capsular polysaccharide phase variation on biofilm formation, motility and gene expression in Vibrio vulnificus
Source: Gut Pathog. 2024 Jul 29;16:40. doi: 10.1186/s13099-024-00620-0 (PMC11287873; doi:10.1186/s13099-024-00620-0)
Supplement: Supplementary file 2 — Supplementary Material 2 [file 13099_2024_620_MOESM2_ESM.docx]

**Figure S1. Total counts for both Tr and Op cells with the passage of incubation time.** Data is expressed as the mean ± SD of at least three independent experiments, with three replicates per trial.


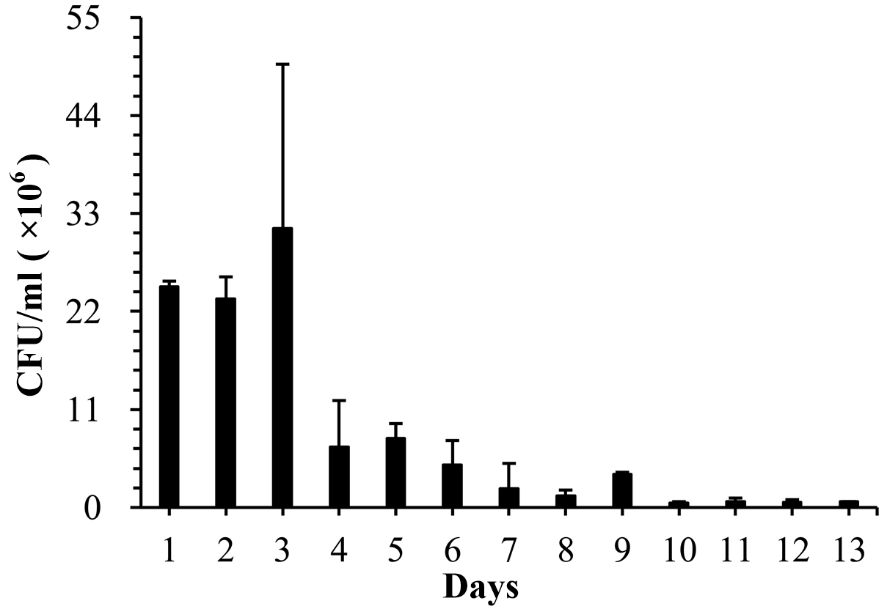


Figure S1
